# Altered Lipid Metabolism in Residual White Adipose Tissues of Bscl2 Deficient Mice

**DOI:** 10.1371/journal.pone.0082526

**Published:** 2013-12-16

**Authors:** Weiqin Chen, Hongyi Zhou, Siyang Liu, Cassie J. Fhaner, Bethany C. Gross, Todd A. Lydic, Gavin E. Reid

**Affiliations:** 1 Department of Physiology, Medical College of Georgia at Georgia Regents University, Augusta, Georgia, United States of America; 2 Department of Chemistry, Michigan State University, East Lansing, Michigan, United States of America; 3 Department of Biochemistry and Molecular Biology, Michigan State University, East Lansing, Michigan, United States of America; University of Graz, Austria

## Abstract

Mutations in BSCL2 underlie human congenital generalized lipodystrophy type 2 disease. We previously reported that *Bscl2* **^−/−^** mice develop lipodystrophy of white adipose tissue (WAT) due to unbridled lipolysis. The residual epididymal WAT (EWAT) displays a browning phenotype with much smaller lipid droplets (LD) and higher expression of brown adipose tissue marker proteins. Here we used targeted lipidomics and gene expression profiling to analyze lipid profiles as well as genes involved in lipid metabolism in WAT of wild-type and *Bscl2^−/−^* mice. Analysis of total saponified fatty acids revealed that the residual EWAT of *Bscl2^−/−^* mice contained a much higher proportion of oleic_18:1n9_ acid concomitant with a lower proportion of palmitic_16:0_ acid, as well as increased n3- polyunsaturated fatty acids (PUFA) remodeling. The acyl chains in major species of triacylglyceride (TG) and diacylglyceride (DG) in the residual EWAT of *Bscl2^−/−^* mice were also enriched with dietary fatty acids. These changes could be reflected by upregulation of several fatty acid elongases and desaturases. Meanwhile, *Bscl2^−/−^* adipocytes from EWAT had increased gene expression in lipid uptake and TG synthesis but not de novo lipogenesis. Both mitochondria and peroxisomal β-oxidation genes were also markedly increased in *Bscl2^−/−^* adipocytes, highlighting that these machineries were accelerated to shunt the lipolysis liberated fatty acids through uncoupling to dissipate energy. The residual subcutaneous white adipose tissue (ScWAT) was not browning but displays similar changes in lipid metabolism. Overall, our data emphasize that, other than being essential for adipocyte differentiation, Bscl2 is also important in fatty acid remodeling and energy homeostasis.

## Introduction

Adipose tissue plays a key role in whole body energy homeostasis. Both obesity (excessive fat) and lipodystrophy (lack of fat) cause dysfunction of adipose tissues which leads to the development of similar metabolic complications including dyslipidemia, diabetes, hypertension and cardiovascular diseases. Congenital generalized lipodystrophy (CGL) is an autosomal recessive disease characterized by a near total absence of body fat from birth or infancy associated with earlier diabetes onset and debilitating metabolic complications [1]–[3]. Mutations in the BSCL2 gene (also called seipin) in humans cause type 2, the most severe form of CGL [4]. Several studies have demonstrated the possible involvement of Bscl2 in adipogenesis, lipid metabolism and lipid droplet biogenesis and maintenance [5]–[9]. However, the function of Bscl2 remains mysterious. Recently, we and two other groups have independently generated *Bscl2^−/−^* mice which display massive loss of white adipose tissues and recapitulate most metabolic disorders of human CGL2 [10]–[12]. In particular, our *in vitro* data using isolated mouse embryonic fibroblasts (MEFs) or stromal vascular cells (SVCs) further revealed that Bscl2 is a novel cell autonomous regulator of cyclic AMP (cAMP)/protein kinase A (PKA) mediated lipolysis and essential for terminal fat cell differentiation [12].

Central fat is more associated with the development of metabolic disorders [13]. Different from *Bscl2^−/−^* mice generated by other groups, we consistently observed about 3% residual EWAT which contained 56% of the DNA in our *Bscl2^−/−^* mice as compared to their wild-type littermates [12]. The residual *Bscl2^−/−^* EWAT displays a browning phenotype with much smaller lipid droplets (LD) and higher expression of brown adipose tissue marker genes [12]. The presence of visceral WAT has also been detected by different techniques in CGL2 patients [14], [15]. Notably, adipose tissue is the main storage compartment for fatty acids with relatively slow turnover time in healthy humans. However, adipose tissue turnover may be influenced by the size of the depot. It is not known whether differences in adipocyte size, or changes in adipose function as observed in lipodystrophy, would affect adipose tissue total or TG fatty acid composition. Therefore, it is critical to understand the molecular events of fatty acid metabolism in residual adipose tissues in order to better control the progression of lipodystrophy.

**Figure 1:**
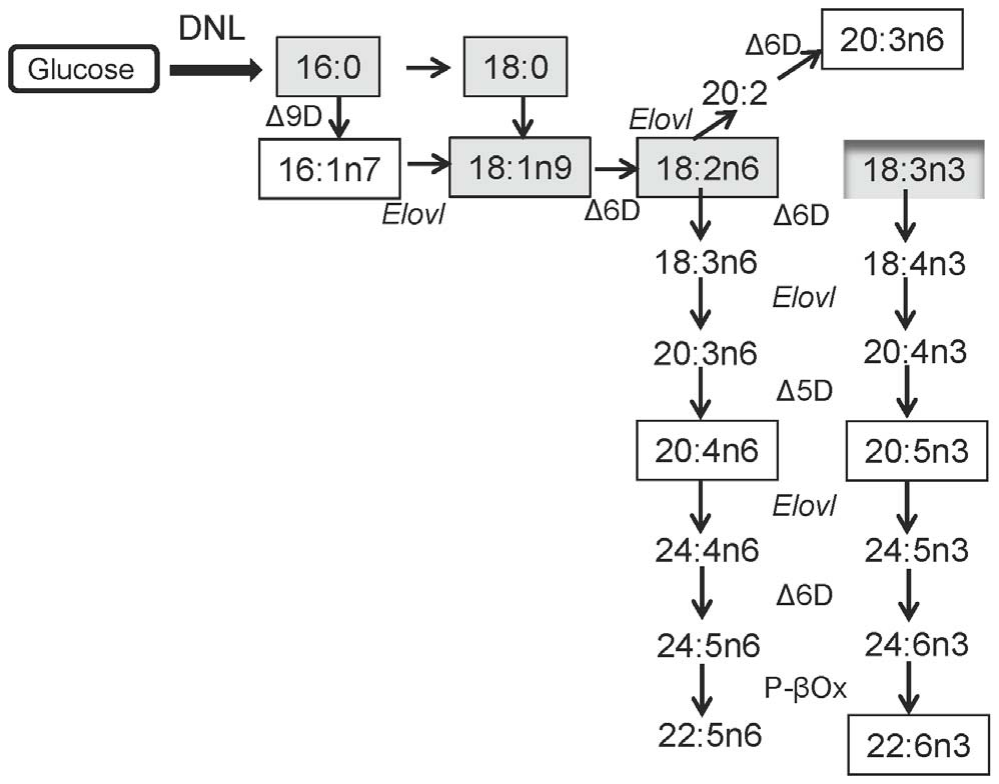
De novo lipogenesis and metabolic pathways of MUFA and PUFAs. Fatty acids are synthesized through de novo lipogenesis (DNL) or converted from dietary palmitic_16:0_, oleic_18:1n9_, linoleic_18:2n6_ and α-linolenic_18:3n3_ acids to long chain unsaturated fatty acids *in vivo* by a series of elongation by elongases (Elovl) and desaturation (Δ5 desaturase (Δ5D/Fasd1), Δ6 desaturase (Δ6D/Fads2), or Δ9-desaturase (Δ9D/Scd1)). Fatty acids that accumulate in animal and human tissues are in solid boxes. Fatty acids derived from normal rodent chow diet are shaded in gray.

To understand the effect of lipodystrophy on adipose tissue fatty acid composition, there are two metabolic routes to be considered: de novo lipogenesis and the polyunsaturated fatty acid (PUFA) remodeling pathways [16]. Saturated fatty acids (SFAs), monounsaturated fatty acids (MUFAs), and PUFAs are synthesized from dietary precursors (glucose, palmitic_16:0_, oleic_18:1n9_, linoleic_18:2n6_, α-linolenic_18:3n3_, eicosapentaenoic [EPA_20:5n3_], and docosahexaenoic [DHA_22:6n3_] acids) through a series of desaturation (Δ5-desaturase [Δ5D], Δ6-desaturase [Δ6D], or Δ9-desaturase [Δ9D]) and elongation (Elovl1–7) reactions (Fig. 1). Using targeted lipidomics and gene expression profiling; here we identified substantial modifications in total fatty acid compositions and glycerolipid species in residual *Bscl2^−/−^* EWAT. The residual *Bscl2^−/−^* adipocytes from both EWAT and ScWAT had marked mRNA upregulation of elongases, desaturases, and TG synthesis enzymes as well as mitochondria and peroxisomal β-oxidation genes. These data suggest that in the absence of Bscl2, the residual *Bscl2^−/−^* adipocytes are still actively mobilizing dietary fatty acids through constant elongation, desaturation, TG remodeling, fatty acid oxidation and energy dissipation to counter uncontrolled lipolysis.

## Materials and Methods

### Ethics Statement

All animal experiments were done using protocols approved by the IACUC at Medical College of Georgia at Georgia Regents University (protocol No: 2012-0462). Mice were maintained under standard 12 h light and 12 h dark cycle at 70°F room temperature and fed with a normal chow diet (Teklad Global 18% Protein Rodent Diet 2018) ad libitum. Efforts were taken to prevent and ameliorate any suffering during the experiments and animals were sacrificed using direct cervical dislocation to avoid effect of anesthesia on lipid metabolism.

### Animals

6–10 week old male *Bscl2^+/+^* and *Bscl2^−/−^* animals were used if not specifically indicated. All animals were sacrificed under nonfasting states at 10:00 am.

### Tissue lipid analyses and thin layer chromatography (TLC)

Tissues were homogenized in standard PBS buffer. Lipids were extracted according to Bligh and Dyer [17]. Total lipids were dissolved in chloroform after being normalized to tissue weights and 5 µl samples or TLC standards (Nucheck, 18-4A and 18-6A) were loaded on Silica Gel 60 to separate the phospholipid (PL), triacylglyceride (TG), diacylglyceride (DG), nonesterified free fatty acid (NEFA), cholesterol (CHO) and cholesteryl ester (CE) fractions by one-dimensional thin-layer chromatography, using petroleum ether-diethyl ether-glacial acetic acid (85∶20∶1) and developed with iodine. For enzymatic analysis of triacylglycerides, total lipids were dissolved in 5% triton X-100 in PBS. Triacylglyceride concentration was measured using a triacylglyceride assay kit (Thermo Scientific) and normalized to tissue weights. Total triacylglyceride amounts were calculated based on total EWAT weights.

### Isolation of adipocytes

Epididymal or subcutaneous white adipose tissues pooled from 3 animals were taken from 6 week old wild-type and *Bscl2^−/−^* mice, minced and digested with 2 mg/mL collagenase type IV in PBS with 2% BSA at 37°C for about 40 min, and the digest was filtered through a 100-µm mesh. Filtrates were then spun at 800 xg. The floating adipocytes were then collected and washed with PBS for RNA extraction and gene expression analysis.

### Isolation and differentiation of mouse embryonic fibroblasts (MEFs)

MEFs were isolated and differentiated exactly as previously described [12].

### Reverse transcription and real time PCR

Total RNA was extracted from cells or tissues with TRIzol (Invitrogen) and reverse-transcribed using MLV-V reverse transcriptase using random primers (Invitrogen). Real-time quantitative RT-PCR was performed on the Strategene MX3005. For tissue gene analysis, data were normalized to 2 house keeping genes (cyclophilin A and β-actin) based on the Genorm algorithm (http://medgen.ugent.be/genorm/) and were expressed as fold changes relative to wild-type controls. For cell gene expression analysis, data were normalized to cyclophilin A and expressed as fold changes relative to wild-type.

### Tissue fatty acids analysis by RP-HPLC

Total tissue lipids were extracted and fatty acids were analyzed as described [18]. Briefly, an aliquot of total lipids from the same amount of tissues was saponified (0.4 N KOH in 80% methanol, 50°C for 1 h). Saponified fatty acids were acidified and extracted with diethyl ether and dissolved in methanol. Saponified free fatty acids were then introduced to the HPLC and fractionated using a YMC J-Sphere (ODS-H80) column, detected using ultraviolet absorbance and evaporative light scatter and quantitated by RP-HPLC [18]. Authentic fatty acid standards (Nu-Chek Prep) were used to generate calibration curves for verification and quantification of fatty acids. We presented data as mole% by dividing the amount of each fatty acid (moles) to the total amounts of detected fatty acids (moles) as previously described [18]. Unsaturation index  =  (mole% x number of unsaturation of each fatty acid)/100 (mole% of total identified fatty acids).

### Mass spectrometry analysis of triacylglyceride and diacylglyceride lipids

Lipids were diluted into isopropanol/methanol/chloroform (4∶2∶1 v/v) containing 20 mM NH_4_Ac and 0.5 µM TG d5-(14:0/16:1/14:0) (Avanti Polar Lipids, Alabaster, Al) as an internal standard. All solvents used were filtered HPLC grade (methanol (MeOH) (J.T. Baker, Phillipsburg, NJ), ammonium acetate and chloroform (EMD Chemical, Gibbstown, NJ). Samples were centrifuged and loaded into Whatman Multichem 96-well plates (Fisher Scientific, Pittsburgh, PA), and sealed with Teflon Ultra-Thin Sealing Tape (Analytical Sales and Services, Pompton Plains, NJ). Preliminary mass spectrometry analysis of each lipid sample was acquired at a range of different dilutions to determine the range at which linearity in response for specific lipids was observed, and to ensure that the ratios of specific lipid ion abundances compared to other lipids within the mixture remained constant [19], [20]. Then, samples prepared at an appropriate dilution were introduced to a Thermo Fisher model LTQ Orbitrap Velos (San Jose, CA) mass spectrometer (San Jose, CA) via a chip-based nano-electrospray ionization (nESI) source (Advion NanoMate, Ithaca, NY) operating in infusion mode. Ion transfer conditions were as follows: heated capillary temperature, 250°C; spray voltage, 1.4 kV; S-lens, 50%. Identification and quantitative analysis of each ion observed was achieved based on accurate mass information from the [M+NH_4_]^+^ molecular ions obtained from triplicate analysis of each sample using the Orbitrap (100,000 K resolution) (1 minute data acquisition periods) in positive ionization mode. Quantitative lipid analysis was achieved by determining peak areas from the high resolution MS spectra using the Lipid Mass Spectrum Analysis (LIMSA) v.1.0 software peak model fit algorithm [21], in conjunction with a user-defined database of hypothetical lipid compounds for automated peak finding and for the correction of ^13^C isotope effects. No attempts were made to quantitatively correct for different ESI responses of individual lipids due to concentration, acyl chain length or degree of unsaturation. Thus, the abundance of DG and TG molecular species at each m/z are reported as the % total lipid area for each class. Characterization of DG and TG acyl chains was achieved using CID-MS/MS product ion scans to observe the neutral loss of R_n_COOH+NH_3_ moieties from their [M+NH_4_]^+^ precursor ions [22], followed by MS^3^ to determine the remaining two fatty acyl groups as RCO^+^ groups [23] (ion isolation window, 1 Da; normalized collision energy, 35 V; activation q, 0.25). Note that characterization of the fatty acyl chains in each lipid was restricted to determination of the total number of carbons and double bonds, and that information regarding acyl chain localization (i.e., Sn1, Sn2 or Sn3), and the specific position of the double bonds was not obtained.

### Statistical analysis

Quantitative data were presented as means ± SD or SEM as indicated. Differences between groups were examined for statistical significance with 2-tailed Student’s *t* test. A *P* value of less than 0.05 was considered statistically significant.

## Results

### The browning *Bscl2^−/−^* white adipose tissues display differential lipid profiles

**Figure 2:**
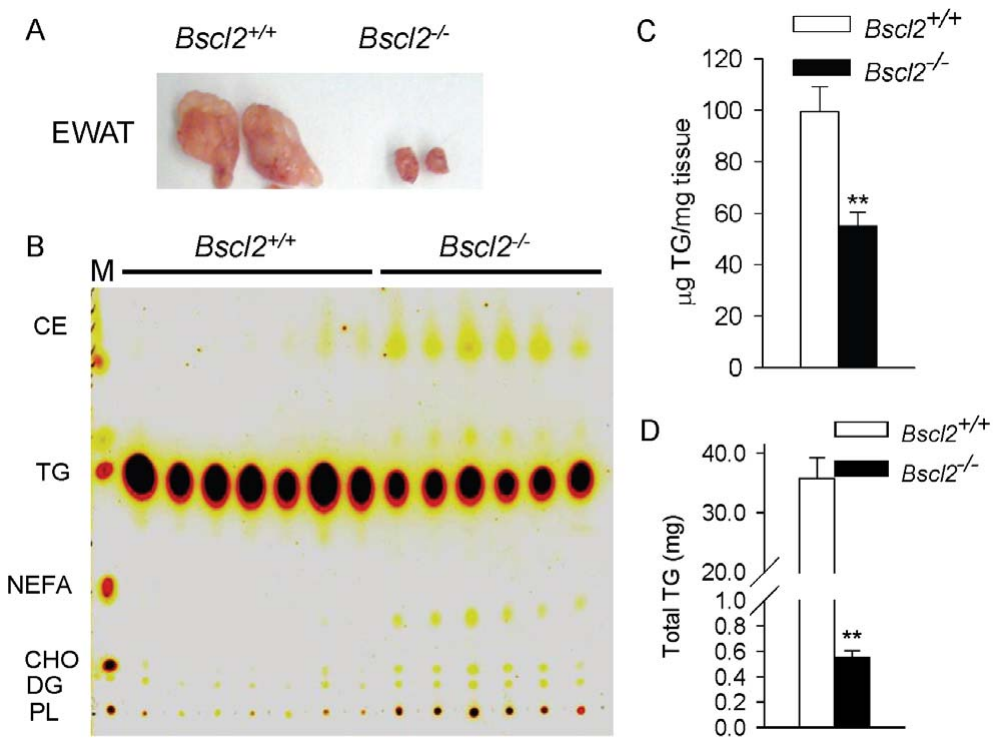
Residual *Bscl2* *^−^* ^*/−*^ EWAT and *Bscl2^+/+^* EWAT exhibit differential lipid profiles. A) Inactivation of Bscl2 in mice causes massive loss of epididymal white adipose tissue (EWAT). B) Thin layer chromatography (TLC) analysis of total lipids extracted from EWAT of male non-fasting *Bscl2^+/+^ and Bscl2^−/−^* mice. M: TLC standards. CE: cholesterol ester; TG: triacylglyceride; FFA: free fatty acid, CHO: cholesterol; DG: diacylglyceride; PL: phospholipid. Total lipids from equal amounts of tissue for each genotype were loaded. B) Enzymatic analysis of EWAT triacylglycerides (TG). Results were presented as µg TG per mg tissue. C) Total EWAT TG contents based on total EWAT weights. n = 6-7 each. **: p<0.005 between two genotypes.

Previously we demonstrated the presence of gonadal white adipose tissues in both male and female *Bscl2^−/−^* mice (Fig. 2A and [12]). The residual EWAT had robust induction of a BAT molecular gene signature including Cidea, Elovl3, Ucp1 and Ppargc1a (encoding PGC-1a), all of which are BAT-selective markers [12]. To characterize the molecular changes in fatty acid metabolism underlying the dramatic browning of the EWAT of *Bscl2^−/−^* mice, we first performed classic thin layer chromatography (TLC) to analyze various lipid classes present in adipose tissues. Surprisingly, if normalized per mg tissue weight, *Bscl2^−/−^* EWAT had decreased levels of triacylglyceride (TG) but increased levels of other lipid classes, including cholesterol ester (CE), nonesterified free fatty acid (NEFA), diacyglyceride (DG), free cholesterol (CHO) as well as phospholipids (PL) (Fig. 2B). Enzymatic analysis confirmed that the TG level per mg tissue in *Bscl2^−/−^* EWAT was 46% less than that in *Bscl2^+/+^* EWAT (Fig. 2C). If calculated based on the total EWAT weights (10±0.9 in *Bscl2^−/−^* vs. 360±87 mg in *Bscl2^+/+^* respectively), the overall TG content in *Bscl2^−/−^* EWAT was only 1.5% of that of *Bscl2^+/+^* EWAT (0.55 0.05 vs. 35.7±3.5 µg) (Fig. 2D). Likewise, the absolute amounts of lipids other than TG would still be lower in *Bscl2^−/−^* EWAT. Nevertheless, *Bscl2^−/−^* EWAT display a shifted lipid profile toward increased abundance of lipids other than TG.

### Altered elongation and desaturation in total fatty acid compositions of visceral white adipose tissues from *Bscl2^+/+^* and *Bscl2^−/−^* mice

**Figure 3:**
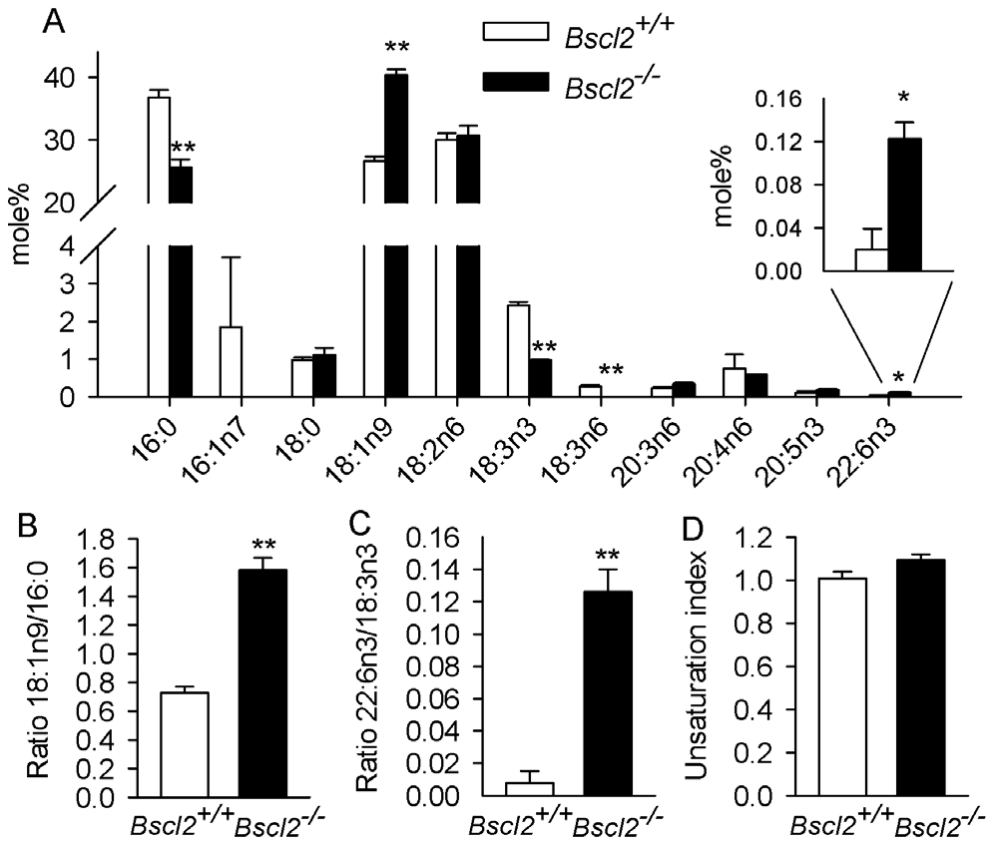
Altered fatty acid compositions suggest increased rate of fatty acid mobilization in residual *Bscl2^−/−^* EWAT. A) Identification and quantification of changes in total adipose tissue saponified fatty acids by RP-HPLC. B) Ratio of oleic_18:1n9_/palmitic_16:0_ acids. C) End product/precursor ratio of DHA_22:6n3_/α-linolenic_18:3n3_ acids. D) Unsaturation index based on the number of double bonds per fatty acyl residue. n = 3 with each sample pooled from EWAT fat pads from 2 animals. *: p<0.05; **: p<0.005.

We next analyzed the total adipose tissue fatty acid compositions of *Bscl2^+/+^* and *Bscl2^−/−^* mice by RP-HPLC. When expressed as mole% of total fatty acids, we observed a decrease in palmitic_16:0_ acid in contrast to an increase in oleic_18:1n9_ acid in *Bscl2^−/−^* EWAT, while no changes in stearic_18:0_, linoleic_18:2n6_, arachidonic_20:4n6_ acids were found between the two genotypes (Fig. 3A). Accordingly, the ratio of oleic_18:1n9_/palmitic_16:0_ acids was significantly increased (Fig. 3B). No palmitoleic_16:1n7_ acid, a potential lipokine derived from adipose tissue that contributes to the regulation of global lipid homeostasis [24], was detectable in *Bscl2^−/−^* EWAT. However, two essential fatty acids, α-linolenic_18:3n3_, γ-linolenic_18:3n6_ acids, were decreased (Fig. 3A) as opposed to increased DHA_22:6n3_ in *Bscl2^−/−^* EWAT. Therefore, the ratio between the major end product of the n3-PUFA synthesis pathway (DHA_22:6n3_, relative to its precursor, α-linolenic_18:3n3_ acid) but not the n6-PUFA synthesis pathway (arachidonic_20:4n6_ acid relative to its precursor linoleic_18:2n6_ acid) was increased, suggesting enhanced n3- PUFA remodeling through elongation and desaturation in *Bscl2^−/−^* EWAT (Fig. 3C). Despite these changes, the unsaturation index (the number of double bonds per fatty acyl residue) remained similar between two genotypes (Fig. 3D).

### Marked changes in molecular species of glycerolipids from *Bscl2^+/+^* and *Bscl2^−/−^* epididymal white adipose tissues

Since approximately 99% of adipose tissue is comprised of glycerolipids including DG and TG, we analyzed glycerolipids by nanoESI-MS, -MS/MS and -MS^3^. 95 TG species were identified with unique masses. Among the 46 TGs present at >0.1% total TG ion abundance in *Bscl2^+/+^* EWAT, most exhibited relative decreases in short or very long fatty acyl chains in *Bscl2^−/−^* EWAT (Table 1). Interestingly, four abundant TGs (TG52:3, TG52:2, TG54:4, TG54:3) were elevated by 14.2%, 41%, 57% and 177%, respectively, in *Bscl2^−/−^* mice vs. wildtype littermates (Table 1). Most of the 49 lower abundant TGs (0.01–0.1% total TG ion abundance) were decreased by more than 50% in *Bscl2^−/−^* EWAT (data not shown). DGs were also present but in much lower amounts and with less diversity of DG molecular species. A total of 18 DGs were identified, with most containing between 32–38 total acyl carbon atoms esterified to the glycerol backbone. 4 DGs were substantially diminished in *Bscl2^−/−^* EWAT including two major species, DG34:2 and DG36:4, along with the minor DG32:4 and DG36:5 species (Table 2). However, the percentage of DG36:2 was 58% higher in *Bscl2^−/−^* EWAT (14.97%±1.6) as compared to *Bscl2^+/+^* EWAT (9.46% ± 0.74). Interestingly, two DGs with very long fatty acyl chains (DG48:1 and DG48:0) were only detectable in *Bscl2^−/−^* EWAT (Table 2). The possible combinations for fatty acyl chains of major TG and DG species were listed in lipid identity excel File S1. Our data suggest that the residual *Bscl2^−/−^* adipocytes were actively involved in remodeling both DGs and TGs.

**Table 1:** Lipidomic analysis of TGs by shotgun mass spectrometry of EWAT from *Bscl2^+/+^* and *Bscl2^−/−^* mice.

TG	*Bscl2^+/+^*	*Bscl2^−/−^*	TG	*Bscl2^+/+^*	*Bscl2^−/−^*
TG38:2	0.28±0.095	0.02±0.004**↓	TG51:2	0.19±0.003	0.17±0.02*↓
TG38:1	0.11±0.04	0.009±0.004**↓	TG52:6	0.33±0.02	0.21±0.04**↓
TG40:3	0.16±0.04	0.025±0.006**↓	TG52:5	3.33±0.14	2.34±0.28**↓
TG40:2	0.15±0.04	0.017±0.007**↓	TG52:4	12.62±0.46	10.55±0.78**↓
TG44:2	0.22±0.05	0.004±0.00**↓	TG52:3	16.2±0.26	18.5±0.11**↑
TG44:1	*0*.18±0.05	0.002±0.001**↓	TG52:2	7.09±0.27	10.01±0.94**↑
TG46:3	0.18±0.03	0.012±0.001**↓	TG53:4	0.21±0.01	0.18±0.03
TG46:2	0.6±0.13	0.03±0.002**↓	TG53:3	0.21±0.01	0.21±0.02
TG46:1	0.47±0.11	0.03±0.004**↓	TG54:7	0.89±0.08	0.24±0.03**↓
TG46:0	0.19±0.04	0.01±0.002**↓	TG54:6	4.59±0.33	3±0.4**↓
TG48:4	0.22±0.02	0.033±0.004**↓	TG54:5	10±0.57	10.6±0.79
TG48:3	0.75±0.09	0.18±0.011**↓	TG54:4	10.2±0.28	16±0.3**↑
TG48:2	1.68±0.2	0.56±0.03**↓	TG54:3	3.17±0.04	8.8±1.08**↑
TG48:1	1.37±0.21	0.46±0.05**↓	TG54:2	0.51±0.01	0.26±0.02**↓
TG48:0	0.7±0.09	0.15±0.02**↓	TG56:8	0.1±0.02	0.04±0.01**↓
TG49:2	0.12±0.001	0.07±0.01**↓	TG56:7	0.24±0.04	0.14±0.04**↓
TG50:5	0.15±0.003	0.1±0.02**↓	TG56:6	0.34±0.05	0.25±0.06**↓
TG50:4	1.3±0.04	0.92±0.12**↓	TG56:5	0.47±0.06	0.34±0.05**↓
TG50:3	4.54±0.17	3.6±0.15**↓	TG56:4	0.5±0.07	0.39±0.05
TG50:2	8.46±0.51	6.44±0.03**↓	TG56:3	0.31±0.07	0.25±0.04
TG50:1	3.12±0.45	2.81±0.24	TG58:5	0.17±0.09	0.12±0.02
TG51:4	0.11±0.01	0.07±0.01**↓	TG58:4	0.22±0.11	0.22±0.03
TG51:3	0.24±0.01	0.20±0.03*↓	TG58:3	0.10±0.03	0.11±0.02

TG species were determined using high resolution ESI-MS and confirmed via product ion scan mode CID-MS/MS and –MS^3^ as described in Methods (n = 3 pooled from 6 animals). Data are expressed as % total TG ion abundance in each genotype. Only the 46 TG species observed at >0.1% total TG ion abundance in *Bscl2^+/+^* EWAT are listed. Data are presented as means ± SD. *: p<0.05; **: p<0.005. Arrows indicate upregulation or downregulation vs. *Bscl^+/+^* EWAT.

**Table 2:** Lipidomic analysis of DGs by shotgun mass spectrometry of EWAT from *Bscl2^+/+^* and *Bscl2^−/−^* mice.

DG	Bscl2^+/+^	Bscl2*^−^* ^/*−*^
DG32:5	0.82±0.59	0.19±0.06
DG32:4	0.29±0.03	0.09±0.02*↓
DG32:3	0±0	0.63±0.96
DG32:2	0.32±0.06	0.27±0.02
DG32:1	1.14±0.6	0.61±0.18
DG32:0	2.08±1.13	0.95±0.15
DG34:3	3.95±1.69	4.31±0.18
DG34:2	32.66±0.82	25.96±0.97*↓
DG34:1	13.73±0.66	15.46±1.79
DG36:5	0.28±0.13	0.07±0.03**↓
DG36:4	11.57±0.88	8.54±0.93*↓
DG36:3	23.2±1.61	24.99±0.59
DG36:2	9.45±0.74	14.97±1.61*↑
DG36:1	0.12±0.11	0.36±0.14**↑
DG38:4	0.12±0.13	1.29±0.16*↑
DG40:8	0.25±0.23	1.02±0.88
DG48:1	0±0	0.11±0.03*↑
DG48:0	0±0	0.12±0.05*↑

DG species were determined using high resolution ESI-MS and confirmed via product ion scan mode CID-MS/MS as described in Methods (n = 3 pooled from 6 animals). Data are expressed as % total DG ion abundance in each genotype. Data are presented as means ± SD. *: p<0.05; **: p<0.005. Arrows indicate upregulation or downregulation vs. *Bscl^+/+^* EWAT.

### Enhanced expression of genes involved in fatty acid modification in *Bscl2* *^−^* ^*/−*^ adipocytes

**Figure 4:**
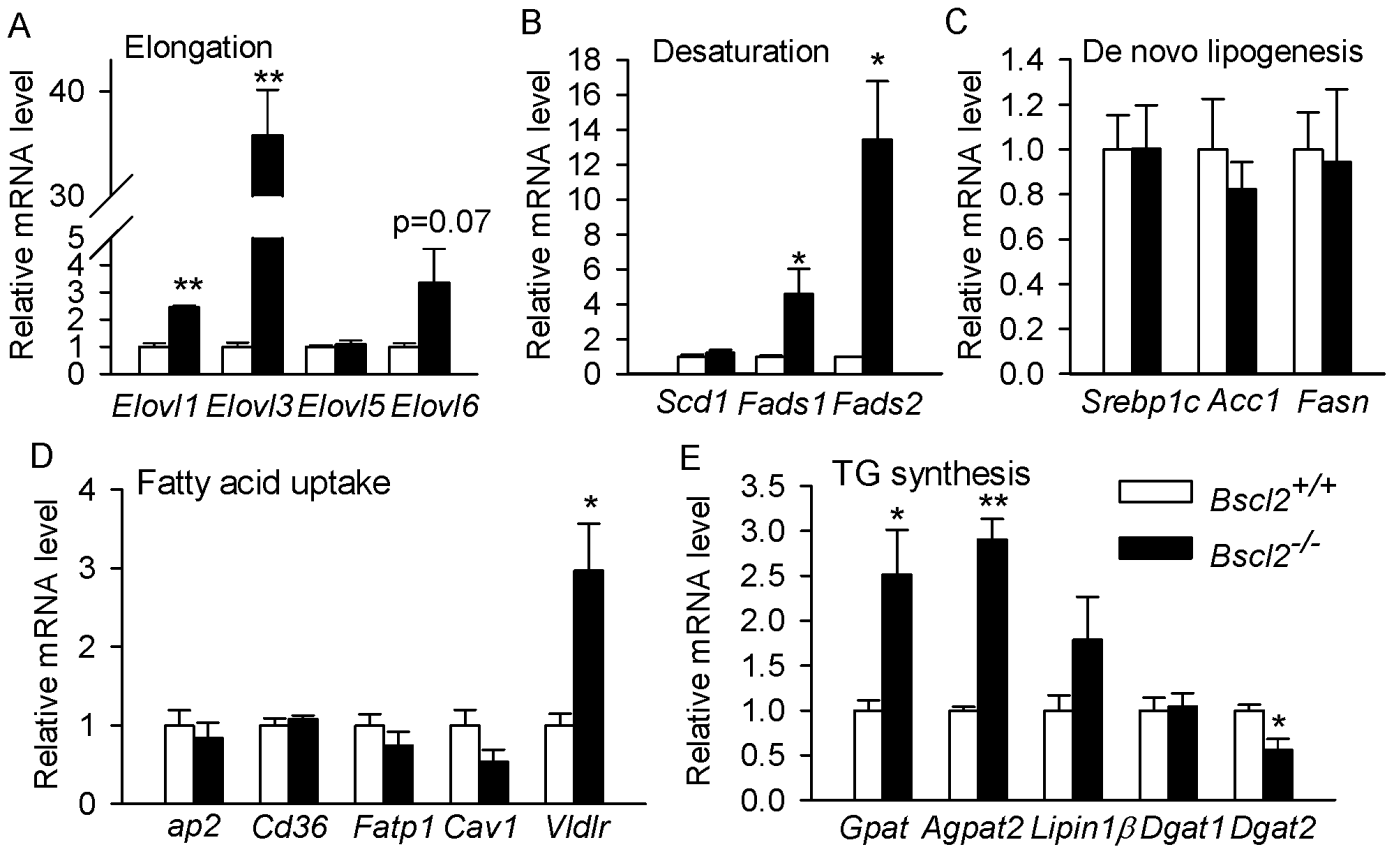
Enhanced expression of key enzymes responsible for elongation, desaturation, and triacylglyceride synthesis but not de novo lipogenesis in isolated *Bscl2* *^−^* ^*/−*^ adipocytes. qPCR analyses of genes involved in elongation (A), desaturation (B), de novo lipogenesis (C), lipid uptake (D) as well as triacylglyceride synthesis (E). Each sample was pooled from 3 6-week-old nonfasting male wild-type and *Bscl2* ^−/−^ mice (*n*  =  4). *: P<0.05; **: p<0.005.

Fatty acid changes in tissues are frequently mirrored by enzyme activities or gene expression differences in fatty acid remodeling enzymes such as elongases and desaturases. We next isolated adipocytes from EWAT of *Bscl2^+/+^* and *Bscl2^−/−^* mice and measured gene expression of elongases and desaturases by qRT-PCR analysis. Elovl1 is commonly regarded as a housekeeping gene in many tissues and is often seen expressed at steady levels [25]. We observed a significant increase in the mRNA levels of Elovl1 (1.56, P<0.05) in adipocytes from *Bscl2^−/−^* mice (Fig. 4A). Confirming the previous results with upregulated Elovl3 in browning *Bscl2^−/−^* EWAT, Elovl3 was increased by 35-fold in *Bscl2^−/−^* adipocytes compared to wild-type adipocytes. No difference was observed in Elovl5 gene expression. Elovl6, an important elongase that drives synthesis of stearic_18:0_ and oleic_18:1n9_ acids from palmitic_16:0_ acid [26], had a tendency toward higher expression that did not reach significance in *Bscl2^−/−^* adipocytes (Fig. 4A). In line with the increased n3-PUFA synthesis in *Bscl2^−/−^* EWAT, the mRNA levels of Δ5D (Fads1) and Δ6D (Fads2) were upregulated by 4.2 and 12 fold, respectively, in *Bscl2^−/−^* adipocytes vs. wildtype adipocytes. Interestingly, despite a higher ratio of oleic_18:1n9_/palmitic_16:0_ acids in *Bscl2^−/−^* EWAT, the major Δ9 desaturase (Scd-1), which catalyzes the synthesis of monounsaturated fats like palmitoleic_16:1n7_ and oleic_18:1n9_ acids, was not different between *Bscl2^+/+^* and *Bscl2^−/−^* white adipocytes (Fig. 4B). We failed to observe any changes in genes involved in de novo lipogenesis, including Srebp1c, Acc1 and Fasn (Fig. 4C). Genes involved in direct fatty acid uptake including ap2, CD36, Fatp1 and Cav1 were not altered. However, the mRNA expression of very low density lipoprotein receptor (Vldlr) was increased by about 3 fold (Fig. 4D), suggesting enhanced uptake of lipoproteins from blood stream by residual *Bscl2^−/−^* adipocytes. Furthermore, the mRNA levels of glycerol phosphate acyltransferase (Gpat) and acylglycerol-3-phosphate acyltransferase 2 (Agpat 2), the critical enzymes responsible for the first two steps of TG synthesis, were increased by 2.5 and 2.9 fold, respectively, in *Bscl2^−/−^* adipocytes. In contrast, we consistently observed an approximately 50% downregulation of Dgat2, the enzyme responsible for the last step of TG synthesis, albeit with no change of Dgat1 gene expression in *Bscl2^−/−^* adipocytes (Fig. 4E).

### Differential upregulation of thermogenic and fatty acid oxidation genes in isolated *Bscl2^−/−^* adipocytes *in vivo* and differentiating *Bscl2^−/−^* MEFs *in vitro*

**Figure 5:**
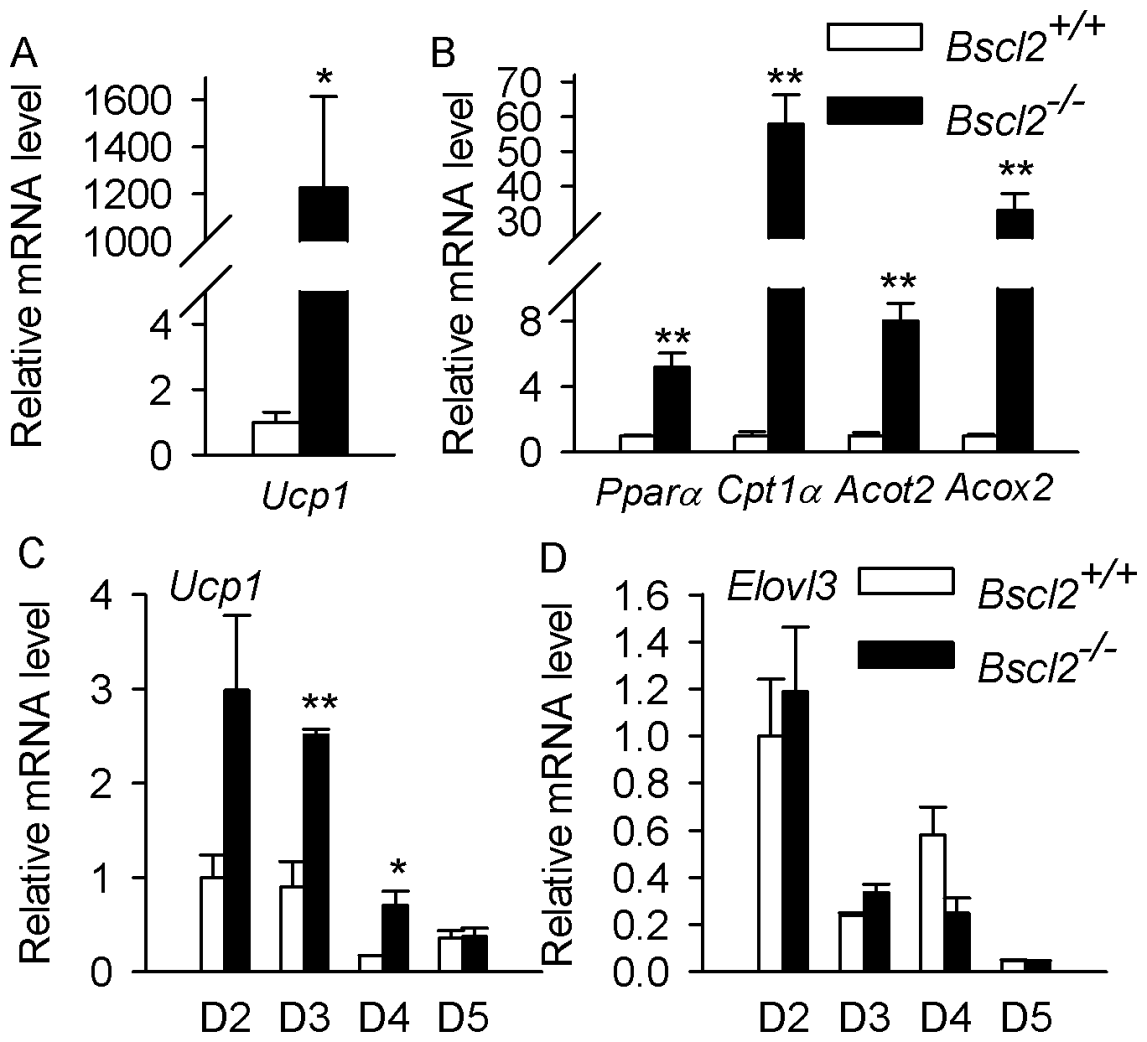
Differential upregulation of BAT specific and fatty acid oxidation genes *in vivo* and *in vitro*. qPCR analyses of BAT specific gene UCP1 (A); genes involved in mitochondrial β-oxidation and peroxisomal lipid oxidation: Pparα and its targeted genes Cpt1α, Acot2 and Acox2 (B) in siolated adipocytes from EWAT of *Bscl2^+/+^* and *Bscl2^−/−^* mice. Each sample was pooled from EWAT obtained from three 6-week-old nonfasting male wild-type and *Bscl2* ^−/−^ mice (*n*  =  4). qPCR analyses of BAT specific gene Ucp1 (C) and Elovl3 (D) in D2 (2 days after hormone cocktail addition to induce differentiation), D3, D4 and D5 differentiating *Bscl2^+/+^* and *Bscl2^−/−^* MEFs. Data were normalized to Cyclophilin A and expressed as relative fold changes as compared to wild-type at D2. *: P<0.05; **: p<0.005.

Accelerated PKA mediated lipolysis underlies the failure of terminal adipocyte differentiation which ultimately leads to massive loss of white adipose tissue in *Bscl2^−/−^* mice. The residual adipocytes in *Bscl2^−/−^* EWAT are still undergoing rampant lipolysis [12]. To accommodate the increased release of free fatty acids, Cpt1α (a critical gene regulating fatty acid β-oxidation), and Cytochrome C (an electron transfer chain protein (CytC)) were upregulated along with a drastic increase in BAT specific Ucp1 gene expression, suggesting increased mitochondrial mediated β-oxidation and energy dissipation through uncoupling in *Bscl2^−/−^* EWAT [12]. Here we confirmed that Ucp1 was specifically upregulated in isolated *Bscl2^−/−^* adipocytes as expected (Fig. 5A). The expression of peroxisome proliferator-activated receptor α (Pparα), the important transcription factor that is activated by lipolytic products and regulates β-oxidation [27], was markedly elevated along with its target genes such as Cpt1α, Acyl-CoA thioesterase 2 **(**Acot2) and Acyl-CoA oxidase 2 (Acox2) in *Bscl2^−/−^* adipocytes vs. *Bscl2^+/+^* adipocytes (Fig. 5B); all of which are involved in fatty-acyl CoA turnover, mitochondria and peroxisomal fatty acid oxidation.

*In vitro Bscl2^−/−^* MEFs ultimately dedifferentiate into nonadipocytes. These cells do not express adipocyte specific transcription factors and their regulated genes [12]. Ucp1 and other BAT specific genes were also unanimously downregulated (data not shown). However, the residual *Bscl2^−/−^* adipocytes *in vivo* are most likely differentiating cells that are undergoing dynamic turn-over. One interesting question remains whether differentiating *Bscl2^−/−^* MEFs *in vitro* also undertake browning with induction of thermogenic and fatty acid oxidation genes. We next focused on D2-D5 differentiating *Bscl2^−/−^* MEFs before most *Bscl2^−/−^* cells turn over to nonadipocytes at D6 and afterwards (based on days after hormone cocktail induction). As shown in Fig. 5C, the expression of Ucp1 tended to be higher in *Bscl2^−/−^* MEFs at D2. At D3 and D4, enhanced expressions of Ucp1 were obvious in *Bscl2^−/−^* differentiating MEFs despite its gradual reduction as differentiation progresses. By D5 this difference disappeared largely due to the dynamic turnover of *Bscl2^−/−^* MEFs. We did not find consistent changes in Elovl3 (Fig. 5D) and fatty acid oxidation genes Pparα and Cpt1α (data not shown). These data agree with a cell autonomous role of PKA activation in stimulating Ucp1 expression. It also suggests other cues may be missing for full browning of differentiating *Bscl2^−/−^* MEFs *in vitro*.

### The residual *Bscl2^−/−^* subcutaneous white adipose tissues were not browning but had similar altered lipid metabolism

**Figure 6:**
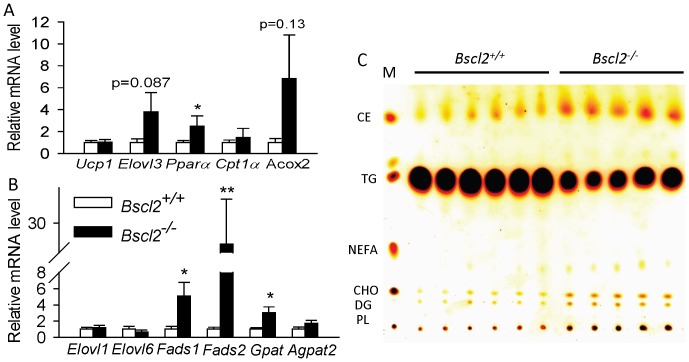
The residual *Bscl2* *^−^* ^*/−*^ subcutaneous white adipose tissues were not browning but had similar altered lipid metabolism. qPCR analyses of BAT specific genes Ucp1 and Elovl3, lipolytic product activated transcription factor Pparα and its targeted genes Cpt1α and Acox2 (A); and genes involved in elongation, desaturation and TG synthesis (B) in isolated adipocytes from ScWAT of *Bscl2^+/+^* and *Bscl2^−/−^* mice. Each sample was pooled from 3-4 6-week-old nonfasting male wild-type and *Bscl2* ^−/−^ mice (*n*  =  4–5). *: P<0.05; **: p<0.005. (C) TLC analysis of total lipids extracted from ScWAT of male non-fasting *Bscl2^+/+^ and Bscl2^−/−^* mice (n = 5–6). Total lipids from equal amounts of tissue for each genotype were loaded.

Subcutaneous white adipose tissue has been shown to have a greater thermogenic capacity and to be more susceptible to cold induced browning than other white adipose tissue depots [28]. Young adult *Bscl2^−/−^* mice retain 30% ScWAT, whose morphology is very similar to *Bscl2^−/−^* EWAT [12]. We next asked whether *Bscl2^−/−^* ScWAT is also browning, and undergoes similar altered lipid metabolism. Suprisingly, in contrast to drastic upregulation of Ucp1 in *Bscl2^−/−^* adipocytes from EWAT (Fig. 5A), Ucp1 mRNA expression was not different between *Bscl2^+/+^* and *Bscl2^−/−^* adipocytes from ScWAT (Fig. 6A) and was even downregulated in *Bscl2^−/−^* animals more than 12 week old (data not shown). A trend toward increased Elovl3 mRNA was detected in *Bscl2^−/−^* ScWAT adipocytes, however, with substantial variability (Fig. 6A). Pparα was slightly upregulated in *Bscl2^−/−^* adipocytes from ScWAT relative to controls, which did not result in a significant increase of downstream Cpt1α and Acox2 genes (Fig. 6A). Analysis of other genes involved in fatty acid elongation and desaturation, as well as TG synthesis, exhibited similar upregulation of Fads1, Fads2 and Gpat genes in *Bscl2^−/−^* adipocytes from ScWAT as observed in EWAT (Fig. 6B). TLC analysis further confirmed that *Bscl2^−/−^* ScWAT had decreased levels of TG in contrast to increased levels of other lipid classes, including CE, NEFA, DG, CHO, and PL when normalized per mg tissue weight, an essentially similar finding as in *Bscl2^−/−^* EWAT (Fig. 2B). These data highlight a fat depot specific browning feature albeit with similar lipid modifications between the two types of the residual *Bscl2^−/−^* WAT.

## Discussion

Adipose tissue dysfunction has a profound impact on whole body lipid metabolism. We for the first time performed lipidomic analysis of a special browning adipose tissue from *Bscl2^−/−^* mice, a mouse model that recapitulates human congenital generalized lipodystrophy type 2 disease. Our findings reveal a substantial modification of fatty acid compositions and glycerolipid species in residual browning *Bscl2^−/−^* EWAT. These changes are correlated with markedly increased expression of genes regulating PUFA synthesis, TG remodeling, mitochondrial and peroxisomal β-oxidation associated with uncoupling. Our data emphasize that the residual *Bscl2^−/−^* adipocytes are actively mobilizing dietary fatty acids through constant lipolysis despite the much reduced adipocyte size and depot, and suggest that the presence of these residual adipocytes may still be able to contribute to whole body energy balance in CGL2 patients.

It is certain that the total content of different lipid classes in *Bscl2^−/−^* EWAT and ScWAT was largely reduced due to the massive loss of adipose tissues. However, when comparing the lipid profiles, we observed a shift towards more abundant phospholipids, NEFA, DG and CE in *Bscl2^−/−^* EWAT and ScWAT as compared to their wild-type counterparts containing predominantly TG (Fig. 2B). This is not surprising considering the marked differences between wild type and *Bscl2^−/−^* WAT in both structure and cell components, with the latter being more compact with much smaller adipocytes infiltrated with immune cell types including macrophages, a phenomena which also happens in lipodystrophy [29].

The fatty acid composition of adipose tissue has been considered as a gold standard to represent dietary fatty acids [30]. Indeed, the total saponified fatty acid compositions of both wild-type and *Bscl2^−/−^* EWAT are enriched with major dietary fatty acids present in the rodent chow diet (Fig. 1 and Fig. 3A). Dietary fatty acids have much greater impact on the fatty acid compositions of *Bscl2^−/−^* EWAT. Oleic_18:1n9_ acid, the most abundant dietary MUFA, was enriched in *Bscl2^−/−^* EWAT (Fig. 3A). The higher proportion of oleic_18:1n9_ acid could also be a result of increased conversion of palmitate to oleate through elongation and desaturation as *Bscl2^−/−^* EWAT has a higher ratio of oleic_18:1n9_/palmitic_16:0_ acids (Fig. 3B). The enrichment of dietary fatty acids in *Bscl2^−/−^* EWAT was also mirrored in its glycerolipids, which contain elevated proportions of TGs (such as TG52:3, TG52:2, TG54:4, TG54:3) and DGs (such as DG36:2) mainly consisting of oleoyl-acyl chains and other dietary fatty acyl chains (highlighted in lipid identity excel File S1). Such enrichment was most likely a result of increased Vldl-TG uptake by *Bscl2^−/−^* adipocytes, an interesting phenomenon which may also help explain lack of hypertriglyceridemia in *Bscl2^−/−^* mice [11], [12]. Notably, we measured the adipose tissue fatty acid compositions in nonfasting animals. As *Bscl2^−/−^* mice are hyperphagic [12], higher dietary intake in *Bscl2^−/−^* mice may contribute to the enrichment of dietary fatty acids. Interestingly, an essential fatty acid, α-linolenic_18:3n-3_ acid was downregulated in *Bscl2^−/−^* EWAT despite overfeeding, largely due to pronounced β-oxidation or enhanced conversion through n3-PUFA pathway to DHA_22:6n3_. Despite a relatively low accumulation of n3-PUFA in adipose tissue lipids, n3-PUFAs especially DHA_22:6n3_ have been found to be less adipogenic and able to prevent excessive growth of adipose tissue and induce mitochondrial biogenesis in adipocytes [31], [32]. Thus far, it is premature to speculate whether such a minimal increased percentage of DHA_22:6n3_ may affect adipocyte differentiation and induce mitochondrial function in the browning *Bscl2^−/−^* EWAT. Nevertheless, these changes implicate that the residual *Bscl2^−/−^* adipocytes are actively metabolizing dietary fatty acids and may still contribute to whole body lipid metabolism.

It is known that the activities of the enzymes involved in both the elongation and desaturation of fatty acids appears to be regulated primarily at the transcriptional level, rather than by posttranslational protein modifications [25]. The increased oleic_18:1n9_/palmitic_16:0_ acids ratio could be explained by increased elongation but not Δ9 desaturation as Scd1 gene expression was not perturbed in *Bscl2^−/−^* animals (Fig. 4A & 4B), suggesting other factors may be involved. Notably, a reduced Δ9-desaturase activity was observed in cultured human *Bscl2^−/−^* fibroblasts which leads to decreased unsaturation ratio [33]. This discrepancy with our result may arise from different cells or tissues measured in each study. In both EWAT and ScWAT of *Bscl2^−/−^* mice, elevated mRNA expression of Δ5D (Fads1) and Δ6D (Fads2) desaturases underlies the increased PUFA remodeling, as both are critical genes for PUFA synthesis pathways (Fig. 1). Elevation of Δ5D and Δ6D genes could be either due to overfeeding in *Bscl2^−/−^* mice as they are known to be nutritionally regulated [25] or through enhanced transcriptional regulation by lipid oxidation associated Pparα activation (Fig. 5B and [34]). Increased Elovl1 was also seen in *Hsl^−/−^* EWAT which was shown to be responsible for increased elongation of palmitic_16:0_ to stearic_18:0_ acids [35]. Elovl3 expression is not only important for TG formation in brown adipose tissue but also highly correlated with increased fatty acid oxidation as well as activation and recruitment of BAT [36], [37]. However, in terms of lipid profile changes in the browning *Bscl2^−/−^* EWAT, our data did not detect an increase in C20-C24 very long chain saturated or monounsaturated fatty acids, the specific products of Elovl3 [25]. These fatty acids could be present at low levels that are below the detection limit by RP-HPLC. Alternatively, the enhanced peroxisomal oxidation in *Bscl2^−/−^* EWAT, as indicated by induction of acyl-CoA oxidase 2 (Fig. 5B), the rate limiting enzyme of peroxisomal very long chain fatty acid (VLCFA) β-oxidation [38], may lead to higher turnover of these fatty acids in the residual *Bscl2^−/−^* EWAT. Nevertheless, DGs with very long fatty acyl chains (DG48:1 and DG48:0) were indeed found in *Bscl2^−/−^* EWAT, underpinning the presence of enhanced fatty acid elongation. Meanwhile, enhanced peroxisomal lipid oxidation also supports the increased conversion of DHA_22:6n3_ which requires β-oxidation of tetracosahexaenoic_24:6n3_ acid in peroxisomes.

Adipocytes with a single deletion of either Dgat1 or Dgat2 were capable of TG synthesis and LD formation [39]. Hence, in spite of a significant downregulation of Dgat2, *Bscl2^−/−^* adipocytes may still be able to synthesize TG normally in the presence of normal Dgat1 expression as highlighted by increased incorporation of major dietary fatty acids into TG species (Table 1). A prominent decrease in proportions of most TGs with either short or very long chain fatty acids can largely be attributed to rampant lipolysis that constantly hydrolyzes TGs in *Bscl2^−/−^* adipocytes. The increased expression of Gpat and Agpat2 suggests the intermediate steps of TG synthesis were probably heightened, which may generate higher proportions of lysophosphatidic acids (LPA) and phosphatidic acids (PA) (Fig. 2A). These data are in line with the previous findings showing increased PA levels in both yeast lacking Bscl2 orthologue Fld1 and drosophila with dSeipin discrupted [40], [41]. Recently, Sim et al demonstrated a critical role of Bscl2 in targeting lipin 1 (PA phosphatase) to the ER, which is required for full Lipin1 function. They showed that differentiating 3T3-L1 cells with simultaneous Agpat2 overexpression and Bscl2 knockdown contained more PA, which led to defective adipocyte differentiation *in vitro* [42]. Given the dynamic turnover of *Bscl2^−/−^* MEF cells undergoing differentiation *in vitro*, our lipidomic analyses of D4 differentiating *Bscl2^−/−^* MEF cells did not yield consistent changes in any lipid species (data not shown). Nevertheless, our findings with consistent mRNA upregulation of Gpat and/or Agpat2 in *Bscl2^−/−^* EWAT and ScWAT suggest Bscl2 could act as an important regulator of lipid synthesis. However, further detailed studies are warranted to elucidate whether such changes in mRNA expression of TG synthesis genes and lipid species are secondary to lipolysis or in fact primary factors that mediate higher lipolysis and/or aborted differentiation in the absence of Bscl2.

In conclusion, our study consists of the first lipidomic analysis of lipodystrophic adipose tissues and reveals dynamic lipid turnover and modification in adipocytes lacking Bscl2. This data further highlights that, in addition to its known essential role in adipocyte differentiation, Bscl2 is also important in fatty acid remodeling and energy homeostasis. Thus, dietary restriction or manipulation in BSCL2 patients may have beneficial effects in alleviating metabolic disorders.

## Supporting Information

File S1 (XLSX)
